# Risk Factors for Metastasis at Initial Diagnosis With Ewing Sarcoma

**DOI:** 10.3389/fonc.2019.01043

**Published:** 2019-10-16

**Authors:** Conglin Ye, Min Dai, Bin Zhang

**Affiliations:** Department of Orthopedics, Artificial Joints Engineering and Technology Research Center of Jiangxi Province, The First Affiliated Hospital of Nanchang University, Nanchang, China

**Keywords:** Ewing sarcoma, metastatic disease, SEER, tumor size, survival

## Abstract

**Purpose:** We aimed to identify potential risk factors predictive of metastasis at initial diagnosis in Ewing sarcoma patients.

**Patients and methods:** We enrolled selected patients diagnosed with Ewing sarcoma between 2004 and 2015 in the Surveillance, Epidemiology, and End Results (SEER) Program database. Demographic and clinical features of patients were analyzed to demonstrate the potential risk factors of distant metastasis at presentation. We utilized descriptive statistics, univariate methods, and a series of regression models to analyze the significance of risk factors. Moreover, we conducted survival analysis in patients with different metastatic sites through Kaplan–Meier analysis.

**Results:** We identified 1,066 cases of Ewing sarcoma and 332 (31.1%) of the patients had metastasis at initial diagnosis. In the univariate logistic regression analysis, patients had higher probability of metastasis at initial diagnosis if they aged between 18 and 59 years old (OR = 1.43; 95% CI, 1.09 to 1.86), had a tumor located in the axial or cranial bones (OR = 1.38; 95% CI, 1.05 to 1.81), or had a tumor over 8 cm (OR = 2.55; 95% CI, 1.66 to 3.89). These three factors were still significant when analyzed in a multivariate logistic regression model or another multivariate logistic regression model controlling for age, location, and tumor size, which had univariate *p* < 0.1. Besides, we found that patients with lung metastasis alone had a better prognosis than patients with bone metastasis alone or with two or more metastatic sites (*p* < 0.01).

**Conclusion:** Ewing sarcoma patients with an age between 18 and 59 years old, a tumor in the axial or cranial bones, and a tumor size over 8 cm had an increased likelihood to have metastatic diseases at initial diagnosis.

## Introduction

Ewing sarcoma (ES) is the second most common primary malignant bone tumor in children and young people, following osteosarcoma (1). Owing to the advance in surgery, radiation, and multidrug chemotherapy in the last few decades, the 5 year overall survival rate of the patients with localized ES has been improved to nearly 75% (2). However, the 5 year survival rate of patients with metastasis is only 20–45%, depending on the metastatic sites (3). It is reported that approximately 25% of ES patients have metastatic diseases at initial diagnosis (4). So far, little is known about risk factors related to higher odds of metastasis at initial diagnosis in ES patients.

Due to the rarity of ES, obtaining adequate cases from our clinical practice to conduct the current research is extremely difficult. Thus, we used the SEER Program database, a commonly used tool to study rare tumors, which provides data from 17 geographically variable cancer registries and involves about 26% of the United States population.

We carried out the current study to identify risk factors of distant metastasis at initial diagnosis in ES patients in both demographic data (age, sex, and race) and tumor characteristics (location and size).

## Materials and Methods

### Ethics Statement

This research was approved by the Ethics Committee of our institution. Since neither human subjects nor personal private information was involved in the data, informed consent from the patients was not required for this study.

### Patient Population

**Figure 1:**
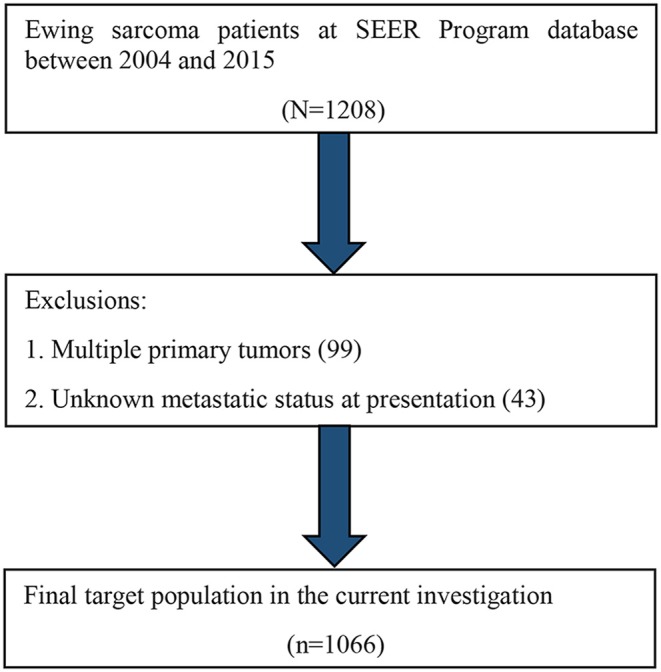
Flow diagram of the target patient population selected from the SEER database.

We identified all the ES cases recorded in the SEER database from 2004 to 2015, utilizing the SEER^*^Stat software (version 8.3.5). We included a total of 1,066 selected cases in this study, as shown in Figure 1.

Among these patients with ES, those with one primary tumor and clear metastatic status at presentation were enrolled in our research. This study focused solely to the bone tumors and extra-osseous ES are not included. Moreover, we excluded patients with multiple primary tumors and those with unknown metastatic status.

We studied demographic features including age, sex, and race. Patient age in the SEER database begins at 0 years and ends at 85 years or more in 5 year intervals. A previous study found that ES patients ≥40 years at diagnosis have a higher possibility to have extraosseous tumors, metastasis, and a lower survival rate (5). Most ES patients are juveniles and there is strong evidence that patients aged 17 years old or less at diagnosis are at reduced risk for death. Thus, in this study, we divided the patients into three age groups of zero to seventeen years old (0–17 years), eighteen to fifty-nine years old (18–59 years), and sixty to eighty-five years old or older (60–85+ years) based on their age at diagnosis. We categorized sex as male or female. The race was characterized as white, black, other (American Indian/AK Native, Asian/Pacific Islander), or unknown.

We also had great interest in tumor-related factors including primary site and tumor size. The primary site in the SEER database is considerably vague, and we could not confirm the explicit bone or the precise site in the bone. Thus, we classified the primary site as the extremity bones (long and short bones of the extremities), axial or cranial bones (pelvis, spine, ribs, mandible, and skull), or unknown sites, similar to what has been done previously (6–8).

We recorded tumor size as a continuous variable. The patients were divided into four size groups of less than 5 centimeters ( ≤ 5 cm), between 5 and 8 centimeters (>5 to 8 cm), over 8 centimeters (>8 cm), or unknown size, according to previous investigations (5, 7, 9).

Distant metastatic sites, including lung, bone, liver, and brain, have been recorded in the SEER database since 2010. Therefore, we utilized the data from 2010 to 2015 to carry out a survival analysis based on different metastatic sites. A total of 152 selected cases were included in the survival analysis, as shown in Figure 4.

### Statistical Methods

We first investigated the total rate of distant metastasis at initial diagnosis among the 1066 patients with ES. Then, we utilized descriptive statistics and univariate methods to determine the percentage of patients with localized disease or metastasis based on the potential risk factors we proposed (age, race, sex, primary site, and tumor size). Lastly, we used several regression models to study the correlation among metastasis at initial diagnosis and a series of demographic and clinical features, including sex, age, race, primary site, and tumor size. Model 1 conducted univariate logistic regression analysis of all the possible risk factors in the 1,066 patients. Model 2 carried out a multivariate logistic regression analysis of all the potential risk factors. Model 3 conducted multivariate logistic regression analysis in variables with univariate *p* < 0.1. We used the log-rank test to evaluate the association between metastatic sites and ES-related survival. *p* < 0.05 was considered statistically significant. We executed all the statistical analysis via SPSS 17.0 software.

### Missing Data

We found missing data in race, primary site, and tumor size. 4/1,066 (0.38%) patients had a missing race variable. 33/1,066 (3.1%) patients had a missing tumor site variable. 263/1,066 (24.7%) patients had a missing tumor size variable. When these predictor variables with missing data were applied in univariate analysis or regression models, we categorized patients with missing data as unknown for statistical analysis.

## Results

**Figure 2:**
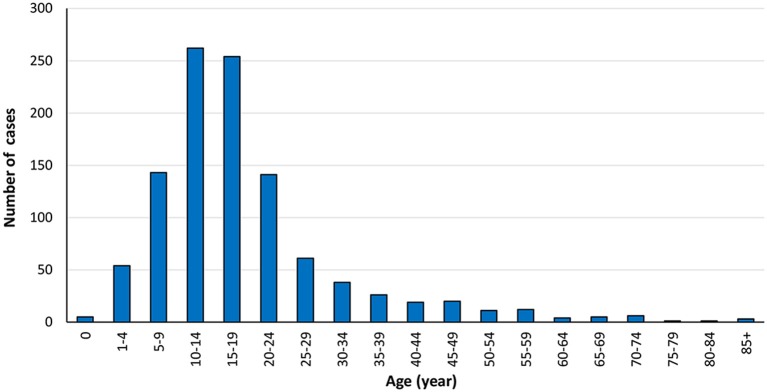
The number of Ewing sarcoma cases from 2004 to 2015 according to age at diagnosis.

**Figure 3:**
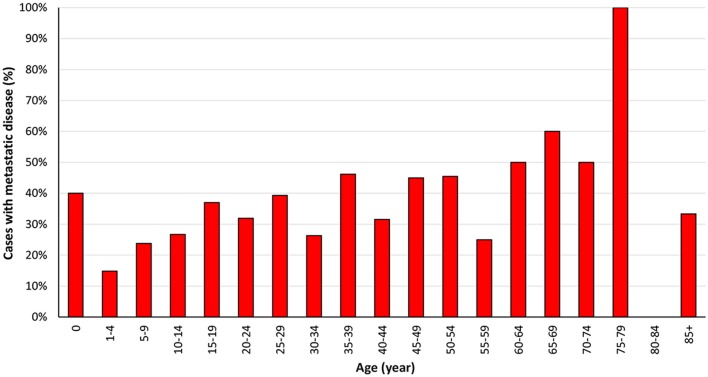
Percentage of Ewing sarcoma cases with metastasis at initial diagnosis from 2004 to 2015 according to age at diagnosis.

We included 1,066 ES cases diagnosed from 2004 to 2015 in the present research. The total proportion of distant metastasis at initial diagnosis was 31.1%, as shown in Table 1. Most of the 1,066 cases occurred in children, adolescents, and young people, which consists with previous research (Figure 2) (7). The ratio of ES patients with metastasis at presentation varied according to the age (Figure 3). Distant metastasis at initial diagnosis was more frequent among patients aged 18–59 years old (35.4%) than patients younger than 18 years old (27.8%) (*p* = 0.006) (Table 2). We also found that axial or cranial primary tumor site and a tumor size larger than 5 cm was related to an elevated rate of metastasis at diagnosis (*p* < 0.001). We found no significant difference in the rate of metastatic disease at diagnosis among patients with different sex (*p* = 0.459) or race (*p* = 0.301).

**Table 1:** Ewing sarcoma with metastasis at diagnosis, 2004 to 2015.

	**No**.	**Metastasis at diagnosis no. (%)**
Total	1,066	332 (31.1)

*No., number; no., number*.

**Table 2:** Univariate analysis of patient characteristics and metastasis at diagnosis with Ewing sarcoma, 2004 to 2015.

**Category**	**No**.	**Metastasis at diagnosis no. (%)**	***p-*value**
Age in years			0.006
0–17	634	176 (27.8)	
18–59	412	146 (35.4)	
60–85+	20	10 (50.0)	
Sex			0.459
Male	673	215 (31.9)	
Female	393	117 (29.8)	
Race			0.301
White	941	294 (31.2)	
Black	40	16 (40.0)	
Other	81	20 (24.7)	
Unknown	4	2 (50.0)	
Location			<0.001
Extremity	472	124 (26.3)	
Axial	561	185 (33.0)	
Unknown	33	23 (69.7)	
Size			<0.001
≤ 5 cm	192	34 (17.7)	
>5 to 8 cm	221	56 (25.3)	
>8 cm	390	138 (35.4)	
Unknown	263	104 (39.5)	

*No., number; no., number*.

The Model 1 univariate logistic regression analysis of all the variables indicated raised likelihood of metastasis at diagnosis among patients aged between 18 and 59 years old (OR = 1.43; 95% confidence interval [CI], 1.09 to 1.86), patients had a tumor located in the axial or cranial bones (OR = 1.38; 95% CI, 1.05 to 1.81), and patients with a tumor size over 8 cm (OR = 2.55; 95% CI, 1.66 to 3.89) (Table 3). The Model 3 multivariate logistic regression analysis, which contained all the variables with univariate *p* < 0.1, also showed increased incidence of metastasis at initial diagnosis among patients aged between 18 and 59 years old (OR = 1.38; 95% CI, 1.05 to 1.82), patients had a tumor located in the axial or cranial bones (OR = 1.42; 95% CI, 1.07 to 1.87), and patients with a tumor size over 8 cm (OR = 2.86; 95% CI, 1.85 to 4.44). The Model 2 multivariate logistic regression analysis of all the variables was carried out to verify the stability of our findings. Model 2 indicated a consistent result with the other two models.

**Table 3:** Odds ratios for risk of presentation with metastatic disease^*^.

**Variable**	**Model 1^a^**	**Model 2^b^**	**Model 3^c^**
Cases included	1,066	1,066	1,066
**Age in years**			
0–17	Ref	Ref	Ref
18–59	1.43 (1.09–1.86)	1.37 (1.04–1.81)	1.38 (1.05–1.82)
60–85+	2.60 (1.07–6.36)	2.03 (0.76–5.43)	2.04 (0.76–5.45)
**Sex**			
Male	Ref	Ref	–
Female	0.90 (0.69–1.18)	0.97 (0.74–1.29)	–
**Race**			
White	Ref	Ref	–
Black	1.47 (0.77–2.80)	1.36 (0.70–2.66)	–
Other	0.72 (0.43–1.22)	0.76 (0.45–1.31)	–
Unknown	2.20 (0.31–15.70)	2.88 (0.39–21.39)	–
**Location**			
Extremity	Ref	Ref	Ref
Axial	1.38 (1.05–1.81)	1.43 (1.08–1.88)	1.42 (1.07–1.87)
Unknown	6.46 (2.99–13.94)	6.04 (2.67–13.68)	6.01 (2.66–13.59)
**Size**			
≤ 5 cm	Ref	Ref	Ref
>5 to 8 cm	1.58 (0.98–2.55)	1.74 (1.07–2.85)	1.76 (1.08–2.87)
>8 cm	2.55 (1.66–3.89)	2.86 (1.84–4.43)	2.86 (1.85–4.44)
Unknown	3.04 (1.95–4.75)	3.14 (1.98–4.97)	3.17 (2.01–5.02)

* *The values are given as the odds ratio, with the 95% confidence interval in parentheses*.

a *Univariate logistic regression analysis of all categorical variables*.

b *Multivariate logistic regression analysis includes all categorical variables*.

c *Multivariate logistic regression analysis includes categorical variables with univariate p < 0.1*.

*Ref, reference*.

Table 4 shows the distributions of distant metastatic sites. The most common ES metastatic sites were lung, followed by bone, liver, and brain. We excluded patients with no specific metastatic sites (*n* = 21), unknown survival months (*n* = 3), metastasis in liver alone (*n* = 1), and metastasis in brain alone (*n* = 1). The remaining cases were used in the Kaplan–Meier analysis. The Kaplan–Meier curve revealed that patients with lung metastasis alone had a better outcome than patients with bone metastasis alone or patients with two or more metastatic sites (*p* < 0.01) (Figure 5).

**Table 4:** The distribution of distant metastatic sites.

**Specific site of distant metastasis**	***n***	**Percentage**
Lung alone	72	45.9%
Bone alone	49	31.2%
Liver alone	1	0.6%
Brain alone	1	0.6%
≥2 sites	34	21.7%

**Figure 4:**
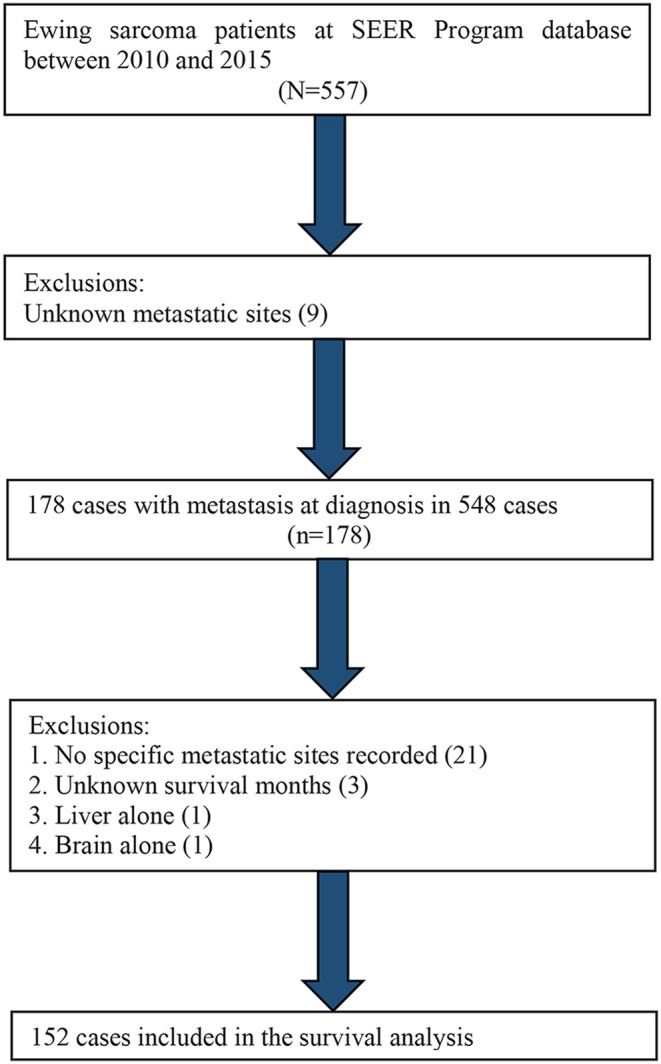
Flow diagram of the patient population selected from the SEER database for the survival analysis.

**Figure 5:**
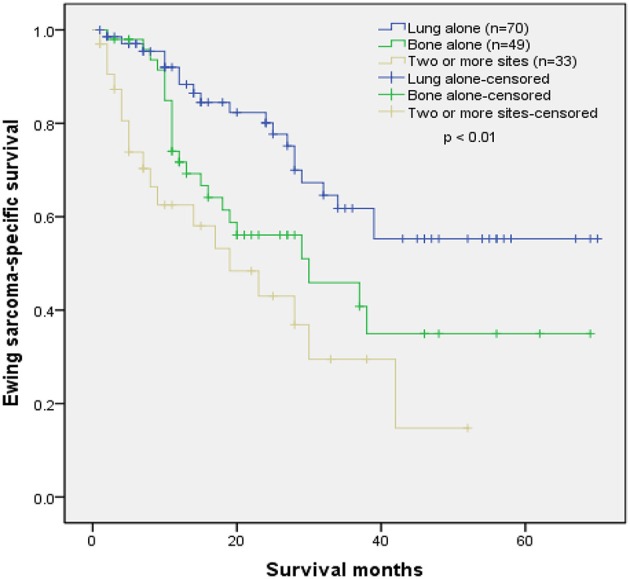
Kaplan–Meier curve of Ewing sarcoma-specific survival according to the metastatic sites.

## Discussion

In this study, we found that 31.1% of ES patients had distant metastasis at initial diagnosis. Age between 18 and 59 years old, axial or cranial tumor sites, and tumor size larger than 8 cm were related to increased odds of distant metastasis at initial diagnosis. Besides, we discovered that patients with lung metastasis alone had better tumor-specific survival rate than patients with bone metastasis alone or patients with two or more metastatic sites.

Previous researches have demonstrated that metastasis at initial diagnosis was an independent predictive factor of poorer overall survival (7, 10–13). Ramkumar et al. found that advanced age, axial tumor location, and larger tumor size were associated with increased odds of detectable metastatic disease at initial diagnosis in patients with Ewing family of tumors (EFT) (14). The current study investigated specifically bone Ewing sarcoma rather than the EFT. To our knowledge, there are few previous researches regarding risk factors for metastasis at initial diagnosis in ES patients. We tried to provide new insights into the predictive factors of distant metastasis at initial diagnosis. Firstly, it included a large sample that was a representative population of the United States. Secondly, we analyzed not only demographic features but also clinical characteristics. Finally, we utilized several multivariate regression models to verify our findings repeatedly. Taken together, we determined several risk factors and therefore helped identify susceptible ES patient groups for metastasis at initial diagnosis.

A few previous researches have identified relevance between older age and a poorer prognosis in ES patients. Karski et al. reported that patients over 40 years old diagnosed with ES were more probable to have metastasis. Moreover, they found that older patients had a lower survival rate (5). Huh et al. also determined patients younger than 10 years old with ES family of tumors had better overall survival rate than older patients (11). In this study, age between 18 and 59 years old was an independent risk factor for metastasis at presentation. Patients younger than 18 years old had lower odds of metastasis at initial diagnosis (*p* < 0.01).

We also determined that an axial or cranial tumor site and tumor size larger than 8 cm contributed to metastasis at initial diagnosis in ES. Some prior researches on ES also showed that tumors in the axial bones and larger tumor size were closely related to a poorer prognosis. For instance, Duchman et al. found that ES patients with metastasis at initial diagnosis, axial tumor site, and tumor larger than 10 cm had lower cause-specific survival rate at 10 years (7). Lee et al. confirmed that older age, metastasis, and larger tumor size were predictive for poor overall survival rate in ES patients (15). The dismal outcomes in these patients could be partly explained by the difficulty in conducting sufficient surgical resection and acquiring proper margins (7, 8, 16). Argon et al. reported that ES originating from the axial bones had a worse outcome than those at the extremities owing to frequent recurrence, fast distant metastasis, larger tumor volume, and difficulties in the surgical intervention (17). Moreover, tumors in the axial bones were usually closer to large vessels, which may elevate the possibility of distal metastatic diseases (18–20). Besides, patients with tumors in the axial bones usually lacked palpable masses or dramatic symptoms. Thus, tumors in the axial bones may also be observed and detected later, which may possibly lead to delayed diagnosis and elevated odds of distant metastasis (7, 8). A larger tumor size also implied increased time before diagnosis and more blood vessels involved. Meanwhile, tumor cells continued to divide uncontrollably over time. These might facilitate metastatic diseases at initial diagnosis due to larger tumor size. In the present study, we merged patients with cranial ES and axial ES into the axial or cranial location category for statistical analysis. The result was similar to previous studies. Cotterill et al. demonstrated that there was a trend for better survival for patients with lung involvement compared with patients with bone metastases or a combination of lung and bone for the ES patients with metastases (13). In this research, we came to a consistent conclusion that patients with lung metastasis alone had a better prognosis than patients with bone metastasis alone or patients with two or more metastatic sites.

Although the present research did not probe into treatment guidance or prognostic factors, our findings did have some important clinical significance. With the awareness of these high-risk factors, doctors can inform certain patient groups about the high possibility of metastasis at initial diagnosis. Patients with high-risk factors might benefit from more frequent and cautious pulmonary surveillance or screening examinations at early stage. Early diagnosis and early treatment could obtain better outcomes. Besides, according to the different metastatic sites, the doctor could partly predict the prognosis of ES patients.

However, the present research had several limitations. Firstly, though the SEER database provided numerous cases to analyze, it did have some inevitable restrictions. We could not verify the diagnostic accuracy of metastasis. Besides, we could not acquire exact information about tumor size or precise location of the tumors. Secondly, we did not investigate socioeconomic factors such as income, poverty, or education status of the patients. Thirdly, we did not examine the survival status in patients with liver or brain metastasis alone. Finally, we did not study the treatment methods or prognostic factors. These were not the goal of the current research, but they represented a crucial part for further exploration.

## Conclusions

In short, the present study demonstrated that age between 18 and 59 years old, tumor located in the axial or cranial skeleton, and tumor size > 8 cm were closely related to a greater likelihood of distant metastasis at initial diagnosis in patients with ES. Additionally, patients with lung metastasis alone had a better prognosis than patients with bone metastasis alone or patients with two or more metastatic sites.

## Data Availability Statement

Publicly available datasets were analyzed in this study. These data can be found here: https://seer.cancer.gov/.

## Ethics Statement

The studies involving human participants were reviewed and approved by the medical ethics committee of The First Affiliated Hospital of Nanchang University. Written informed consent from the participants' legal guardian/next of kin was not required to participate in this study in accordance with the national legislation and the institutional requirements.

## Author Contributions

CY, MD, and BZ designed this study. CY performed the search and collected data. MD and BZ rechecked data. CY performed analysis and wrote the manuscript. All authors reviewed the manuscript.

### Conflict of Interest

The authors declare that the research was conducted in the absence of any commercial or financial relationships that could be construed as a potential conflict of interest.
